# Linking Immuno-Epidemiology Principles to Violence

**DOI:** 10.1186/s12889-022-14472-3

**Published:** 2022-11-18

**Authors:** Anna Sisk, Patricia Bamwine, Judy Day, Nina Fefferman

**Affiliations:** 1 Department of Mathematics, University of Tennessee, 1403 Circle Dr, 227 Ayres Hall, Knoxville, TN 37916 USA; 2 College of Social Work, University of Tennessee, 1618 Cumberland Ave., 401 Henson Hall, Knoxville, TN 37996 USA; 3 Applied BioMath, LLC, 561 Virginia Road, Suite 220, Concord, MA 01742 USA; 4 Department of Ecology & Evolutionary Biology, University of Tennessee, 569 Dabney Hall, Knoxville, TN 37996-1610 USA

**Keywords:** Mathematical model, Public health, Violence exposure, Protective factors

## Abstract

**Background:**

Societies have always struggled with violence, but recently there has been a push to understand violence as a public health issue. This idea has unified professionals in medicine, epidemiological, and psychology with a goal to end violence and heal those exposed to it. Recently, analogies have been made between community-level infectious disease epidemiology and how violence spreads within a community. Experts in public health and medicine suggest an epidemiological framework could be used to study violence.

**Methods:**

Building upon results from community organizations which implement public health-like techniques to stop violence spread, we look to formalize the analogies between violence and infectious diseases. Then expanding on these ideas and using mathematical epidemiological principals, we formulate a susceptible-exposed-infected model to capture violence spread. Further, we ran example numerical simulations to show how a mathematical model can provide insight on prevention strategies.

**Results:**

The preliminary simulations show negative effects of violence exposure have a greater impact than positive effects of preventative measures. For example, our simulation shows that when the impact of violence exposure is reduced by half, the amount of violence in a community drastically decreases in the long-term; but to reach this same outcome through an increase in the amount of after exposure support, it must be approximately fivefold. Further, we note that our simulations qualitatively agree with empirical studies.

**Conclusions:**

Having a mathematical model can give insights on the effectiveness of different strategies for violence prevention. Based on our example simulations, the most effective use of community funding is investing in protective factors, instead of support after violence exposure, but of course these results do not stand in isolation and will need to be contextualized with the rest of the research in the field.

**Supplementary Information:**

The online version contains supplementary material available at 10.1186/s12889-022-14472-3.

## Background

Societies have always struggled with the causes and effects of violence and to a large extent, current methods for curbing violence and its detrimental ripple effects in communities are inadequate. Unfortunately, children and adolescents are often disproportionately impacted by these inadequacies [1]. Each day in the United States, more than 10 adolescents are victims of homicide and over 1,300 are treated for non-fatal assault-related injuries, according to the Centers for Disease Control and Prevention (CDC) [2]. This age group is at a greater risk for poly-victimization, which is experiencing multiple forms of victimization and/or violence. According to The National Survey of Children’s Exposure to Violence, a child who has experienced a physical assault would be five time more likely to have been sexually abused and more than four times as likely to have experienced maltreatment [3]. Furthermore, while the effects of violence reach every demographic, young people in minority communities bear the brunt of the consequences. Homicide is the leading cause of death for Black adolescents and young adults aged 15 to 24 and the second leading cause of death for Hispanic adolescents and young adults in the same age range [4]. The primary way to handle the epidemic of violence our society faces is through the criminal justice system, but recently there has been a drive to treat violence as a disease. With this new view of violence, we can utilize community-based intervention models to better understand and treat violence from a public health perspective.

One recently developed approach focuses on viewing violence as an infectious disease. This idea was based on similarity to patterns of spread in maps of disease and violence outbreaks, such as the appearance of clusters that easily spread to surrounding areas [5]. The intervention-focused organization, CeaseFire, in Chicago, Illinois attempts to prevent violence in high crime areas by implementing intervention/disruption techniques similar to those used to prevent disease spread, such as using contact tracing to find people who are exposed to an act of violence, developing community awareness programs, and both physically and socially distancing individuals at risk of engaging in violence. Thus far, these efforts have achieved a decrease in all shots fired in 4 of the 7 neighborhoods in which CeaseFire has operated [6]. Community-based violence intervention programs can now be seen in American cities like New York, Baltimore, Pittsburgh, Kansas City, Philadelphia, and Oakland, along with international programs in Colombia, United Kingdom, Mexico, Canada, and South Africa [7].

These programs provide preliminary empirical evidence that treating violence as an infectious disease may be an effective approach for violence prevention. This suggests that further efforts to employ the tool kits of infectious disease prevention may also be beneficial. In epidemiology, mathematical modeling of infectious disease dynamics helps complement our understanding from experimental studies. For instance, the susceptible-infected-recovered (SIR) epidemiological model and its variants, have been mathematically modeled to predict how a disease will spread and to test the effectiveness of different interventions, such as vaccines. A recent example of where this work is clearly showcased is the COVID-19 pandemic [8–10]. Thus, experts in public health and medicine have suggested that these approaches could be used to study violence [5].

While mathematical and statistical models have been employed to study the spread of crime, riots, and policing in the past [11–13], more investigation is needed in exploring exposure driven violence detached from criminality and policing. Recently, agent-based modeling has been used to show the effectiveness of community-based violence prevention organizations, like Cure Violence [14]. Although, little work has been done to model violence spread using mathematical epidemiological principles. Specifically, one yet unexplored, class of mathematical models that may be useful for studying violence is Immuno-Epidemiological models. These are mathematically dynamic multi-scale models that link a within-host disease model to an epidemiological population-level model [15]. This integrative approach allows for the bidirectional feedback between the individual and population level to be studied. It is the ideas from this model type we want to implement to study violence. Specifically, we use epidemiological models to explore the spread of violence on the community level and an immune system model to look at the impact that exposure to violence has at the individual level (as described by the change in their propensity to later commit violent acts themselves). By looking at violence through the Immuno-Epidemiological modeling lens we can gain a deeper understanding of the connection between exposure to violence, prevention and mitigation techniques, and the threshold for committing a violent act. Further, by using this model will be able to quickly and efficiently explore different violence prevention methods without always needing to implement costly and time-consuming empirical studies.

In this paper, we focus on building the mathematical structure for an epidemiological model that describes violence spread through exposure. We use this epidemiological model for violence to run some sample simulations and compare the qualitative outcomes of these simulations to the results from in-field studies. These simulations help us, not only confirm that the mathematical epidemiological approach to studying violence has the potential to be realistic and useful, but also explore some general dynamics of our model between violence, exposure, and intervention/disruption techniques and protective factors. To achieve these goals, we will first expand on the initial analogy of violence as an infectious disease by incorporating the individual (host) scale into the framework and formalize the analogy at both scales. Note, we do not suggest this as a pathway by which to address all of the many varied potential causes of, or methods by which to address, violence (e.g., patriarchy, institutional inequalities, intergenerational trauma, etc.), but this does not mean they are without targeted and practical use. Even though mathematical epidemic models do not focus on “solving” the root cause of infectious outbreaks (germs), they still provide critical tools by which to plan mitigative strategies and effective interventions. In this same way, we are proposing a way to quantitively analyze the dynamics of violence spread that occurs through exposure among young people, and the relative effectiveness of potential intervention strategies. 

## Methods

### Analogy

To continue building on the idea of violence as an infectious disease, we formalized and expanded on this analogy. One of the main ways we did this was to include a within-host component. In the fields of psychology, sociology, and social work, it is well known that exposure to violence not only increases an individual’s risk for further victimization, but also for committing violence themselves. Further it is seen that the use of intervention/disruption techniques, like those implemented by CeaseFire, and protective factors, which are resources that help mitigate the effects of risk factors/violence exposure [1], can reduce the propensity for committing violence [16, 17]. Based on these established and understood principles, we build our model to rely explicitly and uniquely on these factors. Thus, we consider that exposure to violence can have deleterious effects on a person's mental health, just as exposure to an agent of disease can lead to an assault on one's physical health. Similarly, as intervention/disruption techniques and protective factors can positively influence mental health, a person's immune system can provide intervention/disruption to the disease progression and promote host health. For example, protection and promotion of host health can be achieved through intervention/disruption techniques for disease spread mitigation such as social distancing, contact tracing, vaccines, medical treatment and prevention, increased sanitation, and awareness programs. Then following the analogy, protection and promotion of mental health can be achieved through interruption/disruption techniques like those mentioned previously or protective factors, such as family and school connectedness, economic relief in forms of tax credits, access to affordable childcare, nutritional assistance programs, mentoring programs for both youths and parents, and therapeutic support [16, 18].

As with any model of a real-world system, some simplifying assumptions are made to build a mathematical representation for the analogy between violence and infectious disease dynamics. For instance, while there are many different forms of violence (verbal/emotional/physical/etc.) we are focusing on physical, reportable acts of violence. This includes, but is not limited to, physical or sexual assaults/abuse, suicide or suicide attempts, homicide or homicide attempts, gun violence, physical bullying, robberies/muggings, or intimate partner violence. Thus, this analogy is not restricted to looking at one specific type of violence; instead, we are interested in exploring the role that poly-victimization plays in violence spread. This model was designed with the assumption it would be representing youths ages approximately 14–17 without severe underlying mental health issues (defined as interfering with the attainment of major life goals [19]), where the illness can manifest as acts violence, as we are focusing on the role that exposure plays in violence propagation.

### Simulations

To understand the dynamics of the model we simulated some basic, example scenarios. The outputs of these simulations helps confirm the model is displaying expected behavior with respect to the scenario explored. Thus, we moved forward with exploring additional system behaviors. Using these simulations, we can investigate different example scenarios and gain some preliminary insight into the feedback between violence exposure, the interruption/disruption techniques, and protective factors. To simulate the model scenarios, it was necessary to solve the system of equations (available in the supplemental information along with compartmental diagrams of the models, S1 and S2), which was done with Python, utilizing the dopri5 solver. The solutions give the time courses of each class in the model, providing a short- and long-term look at the behaviors of the sub-populations under varying parameter values. For more information on the model parameters see the supplemental information, which includes a table (S3) describing each parameter’s interpretation. For the simulations, a population size of $100$ individuals were assumed. The parameter values used, though determined heuristically at this stage, enable the exploration of simulations that provide an alternative way to study the effectiveness of various interventions and the outcomes of different parameter combinations and fluctuations over time. Therefore, these proof-of-concept simulations and example scenarios provide a means to see the value in exploring and experimenting with model simulations to ultimately help reduce the overall effect of violence in a community.

## Results

**Fig. 1:**
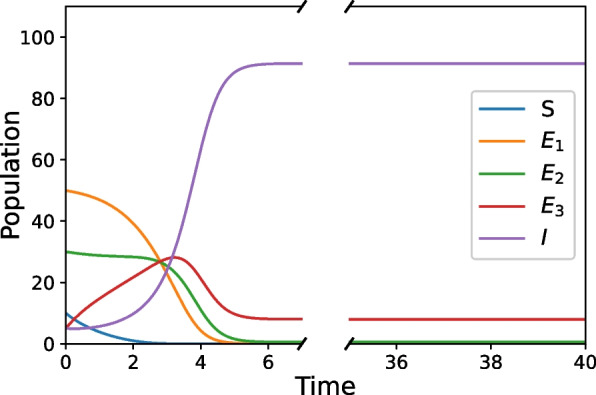
Base case parameter combination that illustrates an example community with a low level of protective factors and intervention/disruption techniques

Figure 1 shows model simulation results in the example population using parameter values chosen as the base case scenario. Subsequent simulation results exploring parameter modifications are then compared back to this base case. We assume there is only a low level of protective factors and intervention/disruption techniques being employed in the base case scenario. Figure 1 shows that in the long term, the Susceptible and Exposed_1_ classes approach a population of zero, the Exposed_2_ population approaches $1$ individual, the Exposed_3_ population approaches $8$ individuals, and the Infectious class approaches a population of $92$. We see the transient dynamics resolve rather quickly (by approximately $t=5$), before nearing the steady state value.

**Fig. 2:**
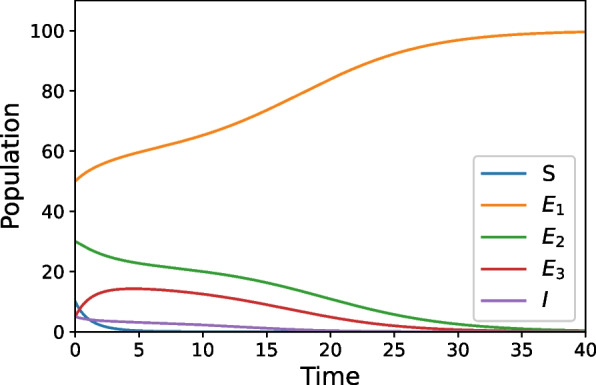
Increases the amount of intervention measures that lower the risk that an exposure will increase violence propensity

**Fig. 3:**
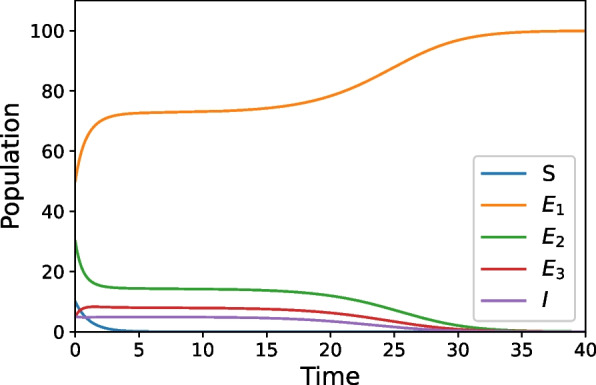
Increases the amount of intervention measures that promote healing after exposure to violence causes an increase of violence propensity

While Figs. 2 and 3 have similar long-term behavior, their transient behavior is quite different. In Fig. 2, the parameters that control the increase of propensity to commit violence after exposure are decreased by half of the values used in the base case, while the other parameters stay at the base case values. In either of these scenarios (Figs. 2 and 3), the entire population is transferring to the Exposed_1_ class and has largely done so by $t=40$. In Fig. 3, we can see a similar behavior by instead varying the parameters that control the amount of intervention measures that promote healing after exposure to violence causes an increase in violence propensity and leaving the other parameters at baseline. For this scenario, the parameters that control healing after exposure needed to be increased approximately five-fold from their baseline values to recreate an outcome comparable to Fig. 2.

**Fig. 4:**
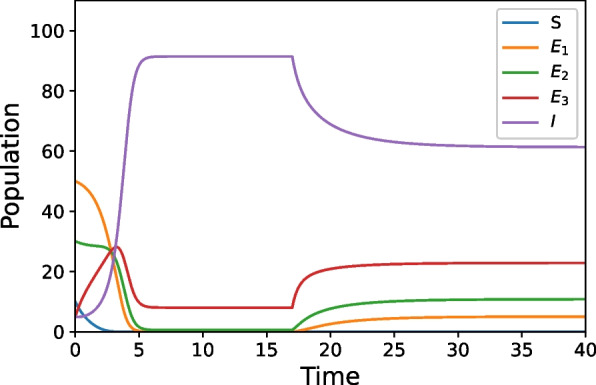
Starts with the same parameters as Fig. 1 and then intervention measures are increased

Figure 4 shows a simulation with dynamic parameter behaviors. In the previous figures the parameters stayed constant throughout the entire run, but in Fig. 4 we introduce a parameter change at $t=17$. The simulation starts with the same parameters as in Fig. 1, but at $t=17$, the parameters that control protective factors, interruption/disruption techniques, and interventions that promote healing after exposure to violence are increased. Note that Fig. 4 approaches its steady state around $t=27$. We observe the transient dynamics level off with a much lower infected population (and respectively higher exposed populations) then it would have without the parameter change. While all four of these simulations are only examples (without any real world data incorporated), we see that the behavior is qualitatively similar to observed real world scenarios [4, 20, 21] and thus exploring these model simulations may provide useful insights into the dynamics of the system and the viability of this approach to violence as a whole.

## Discussion

### Simulations

Our simulations provide an illustration of this approach to show that a mathematical model may be useful in studying real world situations, especially as appropriate data is collected and integrated. While the simulations are preliminary examples, they do illustrate the types of results that could be expected once the model is refined and used in conjunction with other methods. For example, our simulations show that with low levels of protective factors/interruption and disruption techniques violence spreads rapidly through a community, such as what is seen in Fig. 1. This result reflects what may happen in extreme situations when communities are struggling with high rates of violence and have weak community support systems. Further, these simulations also provide a useful strategy for the most effective way to handle violence in a community. Considering the implications of Figs. 2 and 3, we see that investing in violence intervention measures is much more effective then investing in support after exposure. By decreasing in half the impact of violence exposure, the amount of violence in a community goes to zero in the long-term; but to reach this same long-term outcome through an increase in the amount of healing after an exposure, the increase must be approximately fivefold. This implies that investing in programs that give individuals the capacity to cope with violence exposure in a healthy manner (protective factors) are more effective at preventing the spread of violence then programs focused on decreasing a person's propensity to commit violence once that propensity has been increased due to exposure (interruption/disruption techniques). This is, of course, a result we see in epidemiological models: in stopping epidemic outbreaks, it is more effective to prevent people from catching a disease, then treating it once someone is sick.

Further, Fig. 4 illustrates what could happen when interventions are introduced to a community. We see once violence is freely spreading within a community, it may be difficult to get it under control. Even though introducing higher levels of intervention/disruption techniques and protective factors to the run did lower the level of those in the infectious class, there was still high amounts of violence occurring as it approached the steady state. In the supplemental information we explore a simulation (S4) where a community has good levels of intervention/disruption techniques and protective factors, then those levels drastically decrease (for reasons such as a change in funding or priority switch by those leading a community). Although the effects of good intervention measures last for some time in our simulations, eventually the community will end up in the same situation as if there had never been adequate preventative measures. Thus, based on these simulations we can hypothesize that the negative effects of violence exposure have a greater impact in a community then the positive effects of preventative measures. So, these measures should be introduced to a community early and consistently to maximize their effects. Of course, any future results that come from a mathematical epidemiological approach, such as the one here presented, will need to be contextualized within the existing, complementary understanding produced by current research in the field and explored from other violence prevention perspectives.

In general, these insights are in keeping with the qualitative understanding of violence. Empirical case studies such as [4, 20] look at the effectiveness of Cease Fire-like organizations in violence reduction. Statistical analysis of real-world data allowed both papers conclude that using community-based public health prevention measures help to reduce future occurrences of violence. Our model is in qualitative agreement with these data-backed findings that show increasing preventative measures decrease the amount of violence in the communities, as seen in Figs. 2 and 4. Further, studies like [21] look at effectiveness of implementing interventions for those who are hospitalized due to violence. This study shows youths who received these interventions are less likely to commit violence themselves after recovery than those who do not, just as Figs. 3 and 4 predict. Since our models agree qualitatively with data and results from the field, this suggests that the quantitative formulation we have constructed is appropriate for the system. Thus, even though our current simulations are illustrative examples, they help validate our approach to violence prevention as a viable novel contribution to the field.

### Basic reproductive number

An important topic in traditional disease modeling is the basic reproductive number, called ${\mathcal{R}}_{0}$: the number of secondary infections caused by one infectious individual. While the model proposed in this paper is based on concepts from traditional disease models, the basic reproductive number is an aspect where this model diverges from the analogy. For instance, unlike with infectious diseases, exposure to a single violent act by an individual does not directly cause another individual to commit violence, in most cases. Whereas exposure to an individual with an infectious disease will directly cause transmission with some probability. For violence, repeated exposure may be needed to increase a person's propensity to commit violence and that eventually leads to a violent act being perpetrated. Further, though not explicitly explored within our models, there is always the potential for novel emergence of violence from an unexposed individual, which is not possible for infections. Therefore, the traditional concept of the basic reproductive number does not translate well to applications outside of disease modeling. For this reason, in the context of violence we want to re-imagine the concept of the basic reproductive number by developing a more general framework that can work in many different applications. Thus, we propose the idea of the Contributive Number, ${C}_{0}$. For violence, ${C}_{0}$ could describe the damage an act of violence contributes to the collective mental health of a generation. Note, due to the potential for novel violent acts in the absence of exposure, it may be that the contributive number can remain below 1, while violence increases in a population. Understanding this contribution of exposure relative to other factors is part of what such a model can help to characterize and clarify. This idea is one we will continue to explore and develop in the future with respect to its utility for the field and techniques to calculate it mathematically.

### Public health policy

Due to the following well-understood dynamics of violence exposure, we are able to successfully apply a mathematical epidemiological framework and consider violence from a public health perspective: exposure to violence increases the likelihood a person will be further exposed to violence [1, 3], exposure to violence, especially repeated exposures [1], in adolescences increases an adolescent’s propensity to commit violence [17, 18], and protective factors, interruption/disruption techniques, and the passage of time can help decrease a person’s propensity to commit violence after it has been increased due to exposure [4, 16, 18, 20, 21]. As these dynamics closely resemble that of infectious disease spread, our proposed epidemic model for violence is well-grounded. Note, our model does not attempt to tackle the root causes of violence, just as mathematical epidemic models do not propose direct methods to combat germs or how they cause disease, but instead focus on how to limit their spread and impact. Even so, these epidemiological tools are still widely accepted and critical tools in the understanding of outbreak dynamics. Thus, like mathematical epidemic models, our violence model still adds a useful quantitative perspective for prevention and mitigation techniques to the literature.

Having valid quantitative models that capture qualitative understanding allows us to make quantitative predictions for relative efficacy of potential intervention strategies. For those working in the field, like social workers and public health leaders, tools like these can provide results and insight to help determine where effort may be most effective, especially when working with limited resources. For example, the implicit time scale of our model, which focuses on adolescents, aged 14–17, will allow us to explore the most effective age targeted interventions. This has the potential to cause an immediate impact on the public health of communities by reducing the frequency of, and damage caused by, violence. Further, these types of results can also help inform policy makers and community advocates. Mathematical modeling can provide a more time-and-cost-effective approach to gain insight and results to help with allocating and securing funding for violence intervention measures. It can also guide public health policy, law enforcement, and community leaders on the most effective ways to handle violence in communities. This can be especially important if the most effective ways are found to be different from those in current use. An example of this type of result can be seen in our simulations above. It suggest the most effective way to make use of community funding is investing in protective factors, like strengthening economic support for families, changing social norms surrounding violence, providing access to quality low-cost childcare, and offering after school/mentorship programs [18]. Having a general framework in place to study this complex system, such as that proposed in this work, will allow us in the future to customize the model to represent specific communities or types of violence. This view of violence as a multi-scale dynamical system offers a new prospective and mechanism to help stem the epidemic of violence.

### Limitations of the model

Like all mathematical models and simulations, the ones presented here have limitations. The simulations presented in this paper are illustrative in nature as we have not yet incorporated quantitative data from the field. Thus, while the example simulations illustrate the approach and provide a glimpse of how this system works and responds, we are not suggesting that this model provides an accurate representation of what happens in actual cities. Further, the assumptions mentioned earlier also place limits on the model. These assumption stem from decreasing model complexity, especially in the absence of data. In the future, we plan to address these model limitations. We aim to acquire and integrate data on violence from sources such as the National Violent Death Reporting System, the National Crime Victimization Survey, and the FBI’s Uniform Crime Reports. This will allow for better construction of model functional relationships as well as the ability to better estimate model parameters. Although, of course, special consideration will need to be taken into account for any implicit biases that may be embedded in the data. With the inclusion of carefully interpreted data, the model will provide more accurate descriptions of the real-world system and thus better predictions for real communities.

## Conclusion

As violence continues to wreak havoc in our communities, it is clear that viewing violence solely as an issue for the criminal justice system is not effective at preventing the cycle of violence. The promising approach of implementing community-based intervention models to understand and prevent violence has the potential to save countless lives and avoid unnecessary physical and mental trauma. By expanding on the work done by physicians and public health experts, the use of Immuno-Epidemiological modeling can be the next tool to help overcome this societal challenge. While our epidemiological approach does not address the root causes of why violence occurs (just as epidemiology doesn’t address the root causes of pathogens causing infectious diseases) it is still an appropriate and important perspective on violence propagation and prevention. It provides a crucial quantitative tool that can be used to analyze potential intervention and mitigative strategies, and a way to further explore the interplay between violence, exposure, and intervention/disruption techniques and protective factors. Again just as epidemiology is not used in isolation to address an infectious disease outbreak, an Immuno-Epidemiological approach should not be used in isolation to address violence spread. For it to be most effective, it needs to be used in collaboration with traditional violence prevention methods and understood in context with established knowledge in the field.

In this paper, we have formalized the analogy between violence and infectious disease, which expanded the groundwork needed to apply the mathematical tool of epidemiology. Then we discussed a mathematical model for violence spread and explored its potential usefulness by running example simulations. Although, these models are heuristically defined rather than calibrated to data, we nevertheless gained preliminary insights on how different strategies of preventing the spread of violence differed in effectiveness. By comparing the outcomes of our simulations to results from empirical studies, we confirm this approach is an appropriate way to model this system. Further, following the format of the Immuno-Epidemiological modeling structure, once the within host model is integrated, we will be able to study and gain insight into the bidirectional feedback between individuals and their communities when exposed to violence. In conclusion, we see that a public health approach to violence will be an effective way to help curb this epidemic and that mathematical modeling will be an important and useful tool in achieving this goal.

## Supplementary Information

**Additional file 1.**

## Data Availability

The datasets used and/or analysed during the current study are available from the corresponding author on reasonable request.
