# Supplementary material for: Linking Immuno-Epidemiology Principles to Violence
Source: BMC Public Health. 2022 Nov 18;22:2118. doi: 10.1186/s12889-022-14472-3 (PMC9673202; doi:10.1186/s12889-022-14472-3)
Supplement: Supplementary file 1 — Additional file 1. [file 12889_2022_14472_MOESM1_ESM.pdf]

Supplemental Information

Model Parameters

For the One Exposed Class Model the parameter μ governs the effect of initial exposure to violence, representing the rate that susceptible individuals become exposed to violence. Next, ω is the rate at which violence occurs. This parameter could be estimated by looking at the effectiveness of the intervention/disruption techniques. If these are working well then ω would be small; if not, it would be large. It could also be estimated by looking at the literature to determine how poly or re-victimization affects a person's mental health and, thus, propensity to commit violence. Since this parameter is linked to an individual's behavior and mental health, this is where a within-host level model, discussed earlier, could come into play. Finally, β acts as an automatic removal rate, since once the actual act of violence ends there is immediate removal back to the exposed class.

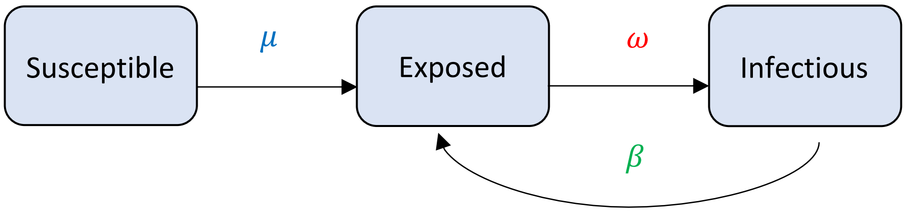

S1 The compartment model diagram for the One Exposed Class model. This is formulated from an SEI epidemiological model to describe violence spread, where the susceptible class has never been exposed to violence; the exposed class has had exposure to violence, but are not committing violence; and the infectious class are actively committing violence. The arrows show movement between the class, while μ, ω, β are the rates that the movements occur.

While the parameters in the Three Exposed Class model are similar to those in the One Exposed Class model, they have important differences. Note, β_1 and β_2 are related to the effectiveness of intervention/disruption techniques, protective factors, and the healing that comes with the passage of time. The more effective these techniques are the faster an individuals' propensity to commit violence will decrease and the faster people will move to a/the less affected exposed classes. While β_3 does move people back one class like the other β parameters, it does not rely on the effectiveness of the intervention/disruption techniques or protective factors like the others do. Once an act of violence is finished, the aggressor immediately moves back to $Exposed_3$ independent of everything. Thus, this parameter functions more as an automatic removal rate then a recovery rate that is dependent on interventions.

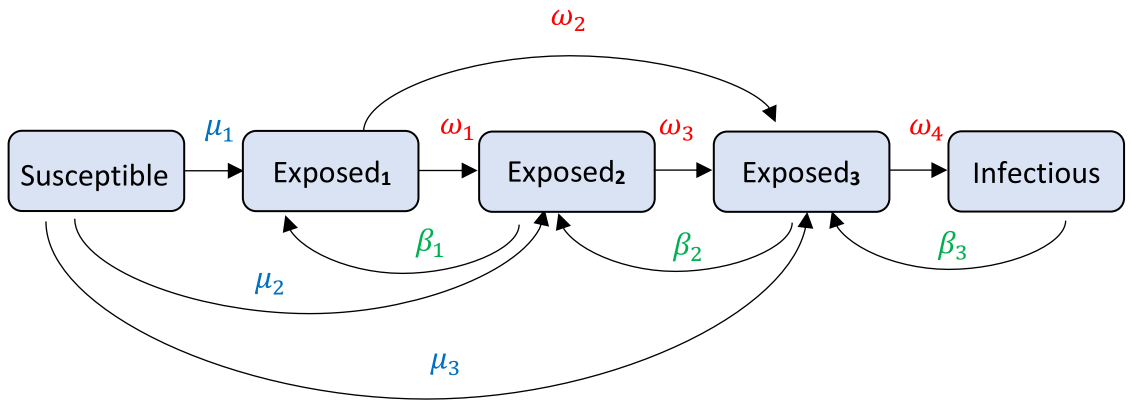

S2 *The compartmental model diagram for the Three Exposed Class model. It is very similar to* **Fig 1**, *except for having three exposed classes and the additional associated parameters. The Exposed$_1$ class has had violence exposure that causes the smallest increase in propensity to commit violence, the Exposed$_2$ class has had violence exposure that causes an intermediate increase in propensity to commit violence, the Exposed$_3$ class has had violence exposure that causes the most severe increase in propensity to commit violence.*

The ω_i parameters look at the effects of exposure to violence, rather than just the exposure itself. Since this is the parameter that moves people from one exposed to the next, it needs to take into account how exposure to violence affects a person's propensity to commit violence. Thus, not every exposure to violence will move an individual to the next exposed class, only exposures that have a significant enough impact to increase the likelihood of committing violence. We would like for this parameter to have two components: one that looks at the 'quality' of the exposure (direct or indirect) and the other that considers the robustness of a person's mental health to an exposure. Thus, with the right support system in place exposure to a certain act of violence would have less of an effect on a person's likelihood to commit violence then if that support is absent. Just as in the One Exposed Class Model, since the ω_i parameters are focused on an individual's response to violence exposure, this is a possible place for a within-host model to come into play. The functional form for this parameter could be described as an exponential function of number of exposures, since the literature reveals that after initial exposure to violence, the likelihood of re-victimization or poly-victimization increases[1]; and increased exposure to violence increases the probability of later committing violence[15 16]. Thus, the values of the ω_i's are taken with $\omega_1 < \omega_3 < \omega_4$ according to an exponential relationship rather than a linear one, for instance. Finally, μ_1, μ_2, and μ_3 focus on the impact that an initial exposure to violence has on a person's tendency to later commit violence, and essentially provides a way for susceptibles to 'skip passed' one (μ_2) or two (μ_3) exposed classes if, for instance, the initial exposure is particularly influential.

Name	Epidemiological	Violence
μ_1	Initial exposure rate that results in a low likelihood of transmission	Rate at which initial exposure to violence increases likelihood of later committing violence (low increase)
μ_2	Initial exposure rate that results in an intermediate likelihood of transmission	Rate at which initial exposure to violence increases likelihood of later committing violence (intermediate increase)
μ_3	Initial exposure rate that results in a high likelihood of transmission	Rate at which initial exposure to violence increases likelihood of later committing violence (high increase)
ω_1	Re-exposure rate	Rate at which exposure to an act of violence increases the propensity of committing violence
ω_2	Re-exposure rate	Rate at which exposure to an act of violence increases the propensity of committing violence (greater increase then ω_1)
ω_3	Re-exposure rate	Rate at which exposure to an act of violence increases the propensity of committing violence
ω_4	Transmission rate	Rate at which an exposure to violence leads to committing an act of violence
β_1	Recovery rate	Decrease in the propensity to commit violence/healing after exposure
β_2	Recovery rate	Decrease in the propensity to commit violence/ healing after exposure
β_3	Removal rate	Cessation (of violence) rate

S3 *Lists the parameters for the Three Exposed Class model, where the second column is the traditional epidemiological definitions and the third column describes the parameters as applied to violence*

Additional Simulation

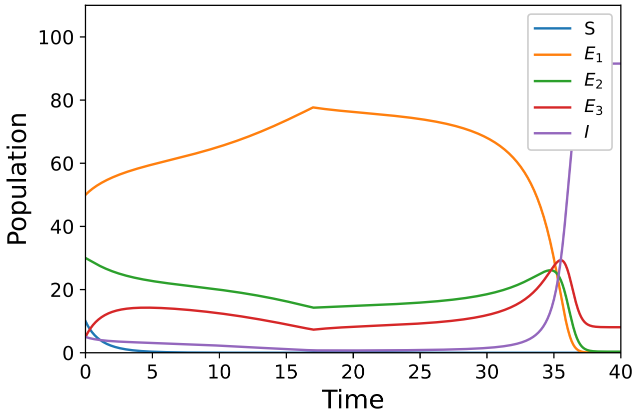

S4 *Starts with the same parameters as* **Fig 2** *and then intervention measures are decreased*

S4 starts with the same parameter combination as **Fig 2** but at $t = 17$, there is an increase in the parameters that control the how propensity to commit violence increases after exposure to violence and a decrease in the parameters that control the amount of intervention measures that promote healing after exposure to violence. We note by approximately $t = 37$ that this run approaches a similar steady state as that in **Fig 1**.

Mathematical Models

One Exposed Class Model:

$$\dot{S} = -\mu S(t)I(t)$$
$$\dot{E} = \mu S(t)I(t) - \omega E(t)I(t) + \beta I(t)$$
$$\dot{I} = \omega E(t)I(t) - \beta I(t)$$

Three Exposed Class Model:

$$\dot{S} = -\mu_1 S(t)I(t) - \mu_2 S(t)I(t) - \mu_3 S(t)I(t)$$
$$\dot{E_1} = \mu_1 S(t)I(t) - \omega_1 E_1(t)I(t) - \omega_2 E_1(t)I(t) + \beta_1 E_2(t)$$
$$\dot{E_2} = \mu_2 S(t)I(t) - \omega_3 E_2(t)I(t) - \beta_1 E_2(t) + \omega_1 E_1(t)I(t) + \beta_2 E_3(t)$$
$$\dot{E_3} = \mu_3 S(t)I(t) - \omega_4 E_3(t)I(t) - \beta_2 E_3(t) + \omega_2 E_1(t)I(t) + \omega_3 E_2(t)I(t) + \beta_3 I(t)$$
$$\dot{I} = \omega_4 E_3(t)I(t) - \beta_3 I(t)$$
